# Electrophysiological evidence for abnormal preparatory states and inhibitory processing in adult ADHD

**DOI:** 10.1186/1744-9081-6-66

**Published:** 2010-10-28

**Authors:** Gráinne McLoughlin, Bjoern Albrecht, Tobias Banaschewski, Aribert Rothenberger, Daniel Brandeis, Philip Asherson, Jonna Kuntsi

**Affiliations:** 1 MRC Social, Genetic and Developmental Psychiatry Centre, Institute of Psychiatry, King's College London, UK; 2 Child and Adolescent Psychiatry, University of Göttingen, Germany; 3 Department of Child and Adolescent Psychiatry and Psychotherapy, Central Institute of Mental Health, Mannheim, Germany; 4 Child and Adolescent Psychiatry, and Center for Integrative Human Physiology, University of Zürich, Switzerland

## Abstract

**Background:**

Attention deficit hyperactivity disorder (ADHD) is a common neurodevelopmental disorder that starts in childhood and frequently persists in adults. Several theories postulate deficits in ADHD that have effects across many executive functions or in more narrowly defined aspects, such as response inhibition. Electrophysiological studies on children, however, indicate that ADHD is not associated with a core deficit of response inhibition, as abnormal inhibitory processing is typically preceded or accompanied by other processing deficits. It is not yet known if this pattern of abnormal processing is evident in adult ADHD.

**Methods:**

The objective of this paper was to investigate event-related potential indices of preparatory states and subsequent response inhibition processing in adults with ADHD. Two cued continuous performance tasks were presented to 21 adults meeting current criteria for adult ADHD and combined type ADHD in childhood, and 20 controls.

**Results:**

The ADHD group exhibited significantly weaker orienting attention to cues, cognitive preparation processes and inhibitory processing. In addition, we observed a strong correlation between the resources allocated to orienting to cues and the strength of the subsequent response strength control processes, suggesting that orienting deficits partly predict and determine response control deficits in ADHD.

**Conclusions:**

These findings closely resemble those previously found in children with ADHD, which indicate that there is not a core response inhibition deficit in ADHD. These findings therefore suggest the possibility of developmental stability into adulthood of the underlying abnormal processes in ADHD.

## Background

Adult ADHD (AD-ADHD) is recognised as a valid and reliable disorder that shares many features with ADHD in childhood [1-3]. Prevalence and longitudinal studies indicate that the cardinal symptoms of ADHD, inattentiveness, overactivity and impulsiveness, persist into adulthood in the majority of cases [4,5]. As some symptoms of ADHD decline in severity throughout development, many individuals who fulfilled symptom criteria for ADHD as children may no longer reach full criteria for ADHD as adults, even though persistence of some symptoms continues to cause significant clinical impairments [6-8].

Several theories postulate deficits in ADHD that have effects across many executive functions, such as response inhibition, attention or working memory, with some data further suggesting that this may be particularly true for a subgroup of ADHD [9]. A meta-analysis confirmed that children with ADHD often perform more poorly than control children on tasks measuring inhibition, vigilance, working memory and planning [10]. Yet, there are many possible explanations for the observed performance deficits [11]. In contrast to performance measures such as speed and accuracy, which provide indirect indices of underlying processes, event-related potentials (ERPs) provide a direct measure of covert brain activity and its precise timing. This is especially true for neuronal processes occurring in the absence of overt behaviour, such as preparatory and inhibitory processes. Hence ERPs can distinguish between different covert processes and elucidate whether behavioural impairments are preceded or potentially caused by certain neuronal deficits.

One of the prominent theories of ADHD proposes that a core deficit of response inhibition underlies the development of broader deficits in executive function, which in turn cause the wide range of dysfunctional behaviours in ADHD [12]. Although, in this model of ADHD behaviour, response inhibition is a vaguely defined concept [13], cognitive-electrophysiological studies have focused on the inhibitory (or no-go) P3 as an index of neurophysiological response inhibition. Studies on childhood ADHD indicate that when inhibitory control deficits have been found, as indexed by the inhibitory P3, they are typically preceded by other processing deficits at the cue stimulus, which suggests that ADHD is not associated with a primary response inhibition deficit [14-21]., Overall, the most consistent cognitive-electrophysiological deficit in childhood ADHD is deficient covert attentional orienting and resource allocation, indexed by reduced P3 amplitude to cues in the cued continuous performance test (CPT-OX) [16-18]. Reduced amplitudes of the subsequent contingent negative variation (CNV) component indicate further deficits related to the expectation of a stimulus, in time processing, motor and non-motor preparation in childhood ADHD [17,18,22]. Further, a recent study indicates that parents of children with diagnosed ADHD, who have a high number of ADHD symptoms (self-report), have attenuated CNV amplitudes to cue stimuli [23]. This abnormal preparatory processing has been interpreted as relating to posterior attentional systems [17], suboptimal state regulation [16] and subcortical generators [23]. Longitudinal ERP work indicates that these orienting, preparation and inhibitory controls deficits all persist from childhood into early adolescence [24].

Few ERP studies have been carried out later in development on AD-ADHD. Studies investigating inhibitory processes in relation to the CPT-OX found that inhibitory no-go-P3 activity was attenuated in adults who reported psychopathology related to childhood ADHD [25] and parents of children diagnosed with ADHD [23]. A study using the stop task reported abnormal attentional processing (as indexed by the auditory N1) prior to altered response inhibition processes in adults with ADHD [26]. However, a recent study failed to find any inhibitory processing problems in a small sample of AD-ADHD probands [27], but this could be due to a small sample size. An investigation focusing on the effects of event rate which affect state regulation found that under a slow condition, the increase in the parietal (go) P3 that is usually observed in healthy controls, was absent in adults with ADHD, with the authors concluding that this suggests state regulation difficulties in ADHD [28]. Finally, a recent study using the standard CPT-OX found inhibitory deficits, as indexed by the no-go P3, in adults who had above threshold scores on either the DSM-IV hyperactive/impulsive or inattentive subscales. The findings did not, however, indicate abnormal parietal P3 components (cue or go) in this sample of adults with ADHD [29].

Given the limited existing ERP data on AD-ADHD, this study aimed to provide a detailed investigation of key ERP indices, previously reported as affected in childhood ADHD, in adults with ADHD, to investigate possible developmental stability in the associated deficits. We used both the standard CPT-OX paradigm [16-18,30,31] and a new version of this task that differs from the original only in that it has a flanker effect on each trial, which is designed to make the task more difficult [23,32]. We hypothesised that the flanker version would give rise to larger case-control differences in the ERP amplitudes in this adult sample [25].The CPT-OX has been shown to be a valid and robust paradigm for the distinct identification of inhibitory processing, response execution and the earlier processes of motor preparation and attentional orienting during a critical but behaviourally silent period [31]. We tested three main hypotheses based on consistently replicated and persistent findings in childhood ADHD. First, that the inhibitory P3 is reduced in adults with ADHD, suggesting persistence of altered response inhibition processing, similar to that seen in childhood ADHD [25,26,28]. Second, that the cue-P3 and the CNV, both precursors to inhibitory processing, are attenuated in AD-ADHD, suggesting developmental stability of the orienting and attentional processes which are consistently found to be abnormal in childhood ADHD [17-19,22]. Our third hypothesis predicts a positive relationship between the cue and no-go-P3 components in both participants with ADHD and normal controls. This would suggest common influences on both the amount of resources allocated to the cue and the strength of inhibitory processing to the following no-go signal.

In addition, we aimed to investigate the functional significance of the no-go-N2. The N2 is thought to specifically relate to inhibitory processing in adults [33,34] but other findings have convincingly related it to the more general process of conflict monitoring [35,36], in that the N2 reflects the level of conflict between the prepared response and the currently required response. We thus investigated the relationship between the go and no-go-N2. A positive relationship between the two components in both case and control groups would suggest that, for the tasks used here, they represent the same process (conflict monitoring) rather than the no-go-N2 specifically representing response inhibition [16].

## Method

### Sample

21 males with ADHD and 20 male controls participated in the study following informed consent. The joint South London and Maudsley and the Institute of Psychiatry NHS Research Ethics Committee approved this study (086/05). All participants were aged 18-40 years, with the ADHD (mean = 32.51, SD = 5.84) and control (mean = 30.00 , SD = 6.51) groups closely matched for age (t(40) = -1.08, p = 0.29). IQ was >79 on the Wechsler Adult Intelligence Scale (WAIS-II) [37]; mean IQ 118 (SD = 10.00) for the ADHD group and 122 (SD = 12.10) for the controls (p = 0.23).

Adults with ADHD were recruited from the National AD-ADHD Clinic at the Maudsley Hospital where they had received the diagnosis from a consultant psychiatrist specialising in AD-ADHD, following in-depth clinical and psychological evaluations. For the purposes of this study, diagnostic criteria for DSM-IV ADHD were applied using clinical interview data for the 18-ADHD symptoms in childhood and adulthood. In addition to the diagnosis from the clinical interview, individuals were only included if either the proband or an informant reported six or more DSM-IV items for both the hyperactive-impulsive and inattentive sub-scales using the Barkley AD-ADHD rating scale for retrospective recall of childhood symptoms [38]; and in addition, six or more inattentive items from the Barkley AD-ADHD rating scale for current symptoms [38]. Participants fulfilled criteria for DSM-IV combined subtype ADHD in childhood and either combined type (n = 17) or inattentive type (n = 4) as adults. The adults with the inattentive subtype were just below threshold on the hyperactive-impulsive subscale (4-6 items, rather than 7 or more).

Exclusion criteria, for the ADHD group, included the presence of an Axis I or II co-morbid psychiatric diagnosis, any previous substance abuse or head injury, taking any psychoactive medication other than stimulant medication, which they were free of for at least 48 hours before testing. All participants were right handed, as determined by preferred writing hand, and had normal or corrected-to-normal vision.

Age and gender matched controls were selected from a database of volunteers at the Institute of Psychiatry, which includes information on psychiatric and medical history. Controls were selected if they had no major psychiatric conditions, substance abuse or previous head injury, but were unselected for ADHD. Self-report data was collected on current and retrospective ADHD symptoms using the Barkley scales [38]. One control had above-threshold symptomatology for the inattentive subtype in the current ratings and two had above-threshold symptomatology for the combined subtype from the retrospective ratings. These individuals were not excluded from the analysis as they were only above threshold on self-reports, they had never sought treatment for their symptoms and did not consider themselves impaired.

### Task and stimuli

The cued CPT was identical to that used in previous studies of childhood ADHD [16-18,30,31] and consisted of 400 black letters (in four identical blocks of 100 letters each and with 11 different letters O, X, H, B, C, D, E, F, G, J and L) subtending approximately 0.5 degrees at the viewing distance of 120 cm. The letters were presented briefly (150 ms) every 1.65 s in a pseudo-random sequence at the center of a computer monitor. The 80 cues (letter 'O') initiated 40 cue-target i.e., 'O' followed by a letter 'X' (go condition), and 40 cue-nontarget sequences i.e., letter 'O' followed by a different letter than 'X' (no-go condition). In 40 cases, a letter 'X' ('distractor-X') was not preceded by a cue 'O' and had to be ignored as well as any other irrelevant letter ('neutral distractors', i.e., all letters except 'O', 'X', and non-target-letters preceded by an 'O'). All cue sequences were pseudo-randomly distributed and occurred with equal probability (10% each); 50% were neutral distractors (all letters except O or X).

The flanker version of the task [23,32] was identical to the original version in the sequence of presentation of stimuli and ratio of conditions but, in this version, each letter was flanked on each side by distractor letters. Letters "X" and "O" were flanked by the incompatible letter O or X, similar to the classic flanker task so that there is a flanker effect on every trial [39], thus turning a O-X sequence into XOX-OXO. Distractor letters were flanked by either "X" or "O". Duration of both tasks was 11 minutes.

Prior to EEG data collection, participants' IQ was assessed using four subtests of the Wechsler Adult Intelligence Scale (WAIS-II): block design, vocabulary, picture completion and similarities [37]. The IQ assessment took 30 minutes in total. During EEG data collection, participants were seated on an adjustable chair in an acoustically shielded, video-monitored room. They were instructed to respond only to cue-target sequences by pressing a button as quickly as possible with the digit finger of their preferred hand. This instruction is assumed to cause a bias toward the go response, when the cue appears, so that stopping this prepared response requires increased inhibitory control or conflict monitoring [40]. The task was practiced and comprehension ascertained prior to task performance. If necessary, participants were told to minimise eye movements or blinks. The two versions of the task were run in a counter-balanced fashion, with another task of 13 minutes duration (not reported here) run in between [41].

### Scoring overt performance

Performance measures in the cued CPT task included target reaction time (MRT, i.e. mean latency of responding in ms after target onset), within-subject variability in reaction times (SD-RT), and the coefficient of reaction time variability (CV, i.e. SD-RT/MRT). MRT and SD-RT were calculated across correctly answered target trials. Hits were characterized by target Xs that were detected between 200 and 1500 ms after stimulus onset. False alarms were responses to letters other than target X. Errors were broken down into subcategories (omission errors, total commission errors, and O-not X commission errors).

### ERP recording and processing

ERPs were recorded with a sample rate of 500 Hz and cut-off frequencies of 0.1-30 Hz via Nihon Kohden Ag/AgCl cup electrodes (impedances kept below 5 kOhm) fixed to the scalp with electrolyte gel at electrode positions, which included the 19 standard electrodes of the 10-20 system, FCz as recording reference, and a ground electrode placed at the forehead using calibrated technical zero baselines and a Neuroscan recording system. Vertical and horizontal electrooculograms (EOGs) were simultaneously recorded from electrodes above and below the left eye and at the outer canthi. The EEG file was analysed using Brainvision Analyzer and was corrected for horizontal and vertical (blinks) eye movements using the Gratton and Coles method [42]. Trials with performance errors or remaining artifacts exceeding ± 100 μV in any channel were rejected from the digitally lowpass-filtered (0.1 to 30 Hz, 24 dB/oct) data before averaging. All trials were inspected visually. Average ERPs were computed separately for each participant in three different stimulus conditions: (1) "go" trials (ERP to Xs preceded by O) (2) "no-go" trials (ERPs to random letters following O), (3) cue trials (ERPs to letter O). All averages were free from residual artifacts and contained a minimum of 20 accepted sweeps (see Table 1). The ERPs were transformed to the average reference for all subsequent computations [43]. Maps of the topographical scalp distribution of electrical brain activity were spline interpolated between the electrode locations. Calibrated zero baselines were used (instead of prestimulus- baseline corrections) to avoid distorting the map topographies [43,44].

**Table 1:** Number of sweeps per stimulus, task and group

	CPT-OX		CPT-OX with flankers	
	Controls	ADHD	Controls	ADHD
**Cue, mean (SD)**	73.70 (6.68)	72.06 (11.42)	75.30 (6.50)	75.94 (5.03)
**Go, mean (SD)**	35.95 (4.58)	34.65 (5.84)	37.90 (2.51)	35.71 (3.92)
**No-go, mean (SD)**	36.90 (3.63)	35.94 (5.44)	37.35 (3.90)	37.41 (3.32)

### Statistical analyses

Two ADHD participants were excluded from the ERP analyses due to excessive movements. ERP amplitudes were restricted to leads and time windows for which effects were expected to be largest, based on previous studies [16,45]. The go-P3 was the largest peak at Pz between 200 and 500 ms; the go-N2 at Fz between 150-300 ms; no-go-P3 at Cz between 200 and 500 ms; no-go-N2 at Fz between 150 and 300 ms; the cue-P3 at Pz between 200 and 500 ms; CNV was the area at Cz between 1300 and 1650 ms. The P3 amplitudes were additionally calculated using mean area amplitudes around the grand mean peak latencies to avoid possible distortions due to a lower signal-to-noise ratio in the ADHD group. The pattern of results remained the same, however, so here we present peak amplitude data in order to remain consistent with the latency analyses of these components. ERP latency data were analysed using analyses of variance (ANOVA). ERP amplitude data were skewed and no transformations were successful (cubic, square, identity, square root, log, 1/square root, inverse, 1/square, 1/cubic). As the data were skewed and it was of interest to investigate task differences as well as group differences, we analysed the data using Generalized Estimating Equations (GEE). GEE models estimate averages, and not the entire distribution of values so it is less restricted by distributional assumptions than other approaches to repeated measures analysis. This approach accounted for the correlation in performance on the two tasks; specifically, an exchangeable correlation structure was assumed to account for the within-subject correlation. This allowed the implementation of a group-by-task interaction to test whether group differences for amplitude of the ERP components would be larger in the flanker CPT task, as predicted. GEE provides unbiased estimates of the marginal effects, even if the assumed correlation structure is misspecified [46,47]. To safeguard a possible misspecification against the variance/covariance matrix, a robust Hubert White sandwich estimator was used to adjust standard errors and hence confidence intervals and p-values [48]. Effect sizes (d) for the ERP amplitudes and performance errors were calculated using the difference of the marginal means from the GEE model, divided by the pooled standard deviation of the raw data. We investigated the relationship between the amplitudes of the ERP components using Spearman's rank correlation coefficient.

For the analysis of performance data, the measures of error rates had pronounced heterogeneity of variance and skewed distributions and transformations were unsuccessful (cubic, square, identity, square root, log, 1/square root, inverse, 1/square, 1/cubic); analyses of these data were therefore performed using GEE models. MRT, SD-RT and CV were analysed using repeated-measures ANOVAs with age as a covariate. Time-on-task effects on RT data were investigated by comparing RT in equal length quartiles over the course of the task in a repeated measures ANOVA. We investigated the relationship between the performance measures using Spearman's rank correlation coefficient.

Although IQ did not differ between groups, initial analyses were run with IQ as a covariate, which confirmed IQ effects as non-significant. IQ was therefore not included in subsequent analyses. A significance level of *p *< 0.05 (two-tailed) was adopted throughout the analyses, with trends (*p*≤09) also reported for predicted effects. We initially included age as a covariate in all analyses and only report it when it was significant, as we dropped it from the analyses otherwise.

## Results

All analyses remained stable when the control participants who retrospectively reported high numbers of ADHD symptoms were excluded (data not shown). We additionally re-ran all analyses excluding the ADHD participants who had subthreshold hyperactive-impulsive symptoms in adulthood and again the results did not change (data not shown). Further, these participants were not outliers on any of the ERP or performance variables.

### Performance measures

Repeated-measures ANCOVA of the reaction time measures indicated no significant effects of age as a covariate, so it was subsequently dropped from analyses, [MRT: F(1, 37) = 3.20, p = 0.08; SD-RT: F(1, 37) = 1.69, p = 0.20; F(1, 37) = 0.60, p = 0.44] nor were there group-by-task interactions for MRT [F(1,39) = 0.36, p = 0.55], SD-RT [F(1,39) = 0.16, p = 0.70] or CV [F(1,39) = 0.20, p = 0.66]. There were, as predicted, significant group differences on MRT [F(1, 39) = 11.90, p = 0.001, d = 1.09], SD-RT [F(1,39) = 20.59, p < 0.001, d = 1.46] and CV [F(1,39) = 19.65, p < 0.001, d = 1.46], with the ADHD group being slower and more variable than the control group (see Table 2). No task differences emerged for any of the variables: MRT [F(1,39) = 0.01, p = 0.93, d = 0.04], SD-RT [F(1,39) = 0.53, p = 0.47, d = 0.32] or CV [F(1,39) = 0.91, p = 0.35, d = 0.30].

**Table 2:** Measures of overall performance in both tasks

	CPT-OX		CPT-OX with flankers	
Mean (SD)	Controls	ADHD	Controls	ADHD
**MRT**	371.43 (80.45)	473.54 (119.78)	377.76 (55.40)	468.79 (106.54)
**SD-RT**	71.86 (32.44)	138.85 (65.81)	69.13 (36.18)	129.16 (62.21)
**CV**	0.19 (0.06)	0.29 (0.11)	0.18 (0.07)	0.27 (0.09)
**Total commission errors**	0.1 (0.31)	0.57 (0.98)	1.0 (1.34)	0.57 (1.12)
**O-not-X commission errors**	0.45 (0.60)	1.33 (1.56)	0.45 (1.00)	2.19 (4.74)
**Omission errors**	0.6 (1.39)	3.23 (4.24)	0.45 (0.69)	2.76 (3.35)

MRT: mean reaction time in milliseconds; SD-RT: within-subject variability in RTs in milliseconds; CV: coefficient of variation (SD-RT/MRT)

Repeated measures ANOVA indicated a main effect of time on task for MRT in the CPT-OX [F(1, 38) = 7.60, p < 0.01, d = 0.90] with no group by time interaction [F(1, 38) = 0.92, p = 0.34]. For SD-RT in the CPT-OX, no main effect of time on task emerged [F(1, 38) = 0.50, p = 0.48, d = 0.22]; however, there was a time by group interaction, with more variation in levels of SD-RT (increases and decreases) in the ADHD group over the course of the task [F(1, 38) = 5.28, p = 0.03]. For the CPT-OX with flankers, no main effect of time on task emerged for MRT [F(1, 39) = 1.78, p = 0.19, d = 0.43], nor was there a group by time interaction [F(1, 39) = 0.00, p = 0.99]. Similarly, for SD-RT in this task, no main effect of time on task emerged for MRT [F(1, 39) = 2.29, p = 0.14, d = 0.49], nor was there a group by time interaction [F(1, 38) = 0.31, p = 0.58].

GEE analysis indicated no significant interaction between group and task for commission errors [z = 0.28, p = 0.78], commission errors of the O-not-X type [z = -1.03, p = 0.31] or omission errors [z = -0.38, p = 0.70]. No significant group differences emerged for commission errors [z = -0.40, p = 0.69, d = 0.08] or commission errors of the O-not-X type [z = 1.34, p = 0.18, d = 0.32]; however, there were very few commission errors (Table 2) so this should be interpreted cautiously. Additionally, the medium effect size for difference in number of commission errors of the O-not-X type suggests that the ADHD group may be abnormal in performance measures of inhibitory control, although we did not detect a significant group difference. The analyses did, however, indicate a significant group difference for omission errors [z = 3.33, p < 0.001, d = 0.99]. We did not obtain any significant differences between the tasks in number of errors [commission errors: z = 0.90, p = 0.37, d = 0.20; commission errors O-not-X type: z = 0.98, p = 0.33, d = 0.18; omission errors: z = -0.72, p = 0.47, d = 0.11].

### Relationship between speed and accuracy

In the original CPT-OX task, Spearman's correlations showed that no significant correlations emerged for MRT and O-not-X commission errors. For the CPT-OX, the direction of correlations was negative in the control group [r = -0.29, p = 0.21] and in the ADHD group [r = -0.16, p = 0.49]. Similarly, in the CPT-OX with flankers, a negative correlation emerged between MRT and O-not-X commission errors for both the control group [r = -0.28, p = 0.24] and the ADHD group [r = -0.24, p = 0.30], indicating a similar effect of inter-individual speed-accuracy trade-off in both groups.

In the original CPT task, a significant positive correlation emerged for MRT and number of omission errors for the ADHD group [r = 0.43, p = 0.05], but not for the control group [r = -0.04, p = 0.88], which indicates that a lower accuracy was accompanied by slower reaction times (RTs) in the ADHD group. Similarly, in the flanker version, lower accuracy was again significantly associated with slower RTs in the ADHD group [r = 0.65, p = 0.002], but not in the control group [r = 0.07, p = 0.76].

### ERP parameters

**Figure 1:**
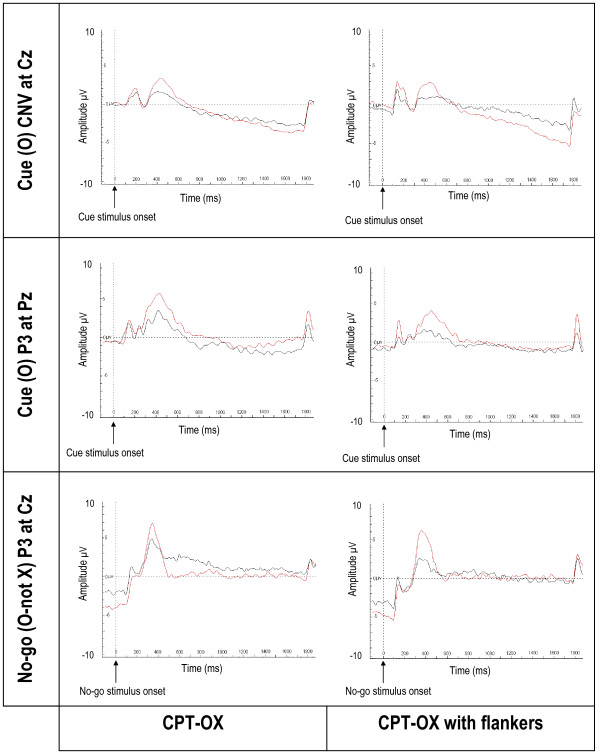
**Grand mean ERPs to cue and no-go stimuli, separately for the control group and the ADHD group**. Red line represents controls; black line represents participants with ADHD. Ticks on horizontal axes represent 100 ms.

The groups did not differ significantly in their cue-P3 latency [F(1,38) = 0.01, p = 0.91, d = 0.04] for the CPT-OX, whereas a significant group difference emerged in the flanker version [F(1,38) = 6.35, p = 0.04, d = 0.82], with the ADHD group activating the preparatory process earlier than the control group (Table 3; Figure 1). For the CPT-OX, the groups did not differ significantly in the latency of the inhibitory components: P3 [F(1,37) = 1.37, p = 0.17, d = 0.39]; N2 [F(1,34) = 0.79, p = 0.38, d = 0.30] or the executory P3 [F(1,38) = 0.02, p = 0.90, d = 0.04] and N2 [F(1,38) = 0.53, p = 0.47, d = 0.24] (Table 3). Similarly, for the CPT-OX with flankers, the groups did not differ significantly in the latency of the inhibitory components: P3 [F(1,37) = 0.16, p = 0.69, d = 0.14]; N2 [F(1,38) = 1.88, p = 0.18, d = 0.45] or the executory P3 [F(1,37) = 0.68, p = 0.60, d = 0.26] and N2 [F(1, 37) = 0.51, p = 0.48, d = 0.24] (Table 3).

**Table 3:** Amplitude and latency of ERP components

			Amplitude		Latency	
			**Controls**	**ADHD**	**Controls**	**ADHD**

	**Cue**	**P3 (Pz)**	6.45 (2.53)	4.43 (1.91)	396.88 (47.55)	398.85(63.11)
		**CNV (area at Cz)**	-3.21 (2.49)	-2.09 (1.22)	#	#
**CPT-OX**	**Go**	**P3 (Pz)**	7.64 (3.49)	5.89 (2.60)	338.09 (33.23)	336.35(49.97)
		**N2 (Fz)**	-3.68 (3.73)	-1.82 (3.32)	253.32 (35.51)	261.92(38.22)
	**No-go**	**P3 (Cz)**	7.45 (3.71)	5.38 (2.63)	341.99 (31.13)	360.68(49.56)
		**N2 (Fz)**	-4.44 (3.44)	-2.88 (2.57)	240.43 (28.38)	250.59(25.80)

	**Cue**	**P3 (Pz)**	5.49(1.92)	3.41(1.63)	407.81 (67.84)	343.97(95.41)
		**CNV (area at Cz)**	-3.72 (2.25)	-1.88 (1.30)	#	#
**CPT-OX with flankers**	**Go**	**P3 (Pz)**	7.71 (3.14)	6.90 (4.17)	364.65 (49.74)	374.13(60.57)
		**N2 (Fz)**	-3.67 (2.39)	-2.95 (3.14)	242.38 (33.01)	251.09(41.96)
	**No-go**	**P3 (Cz)**	7.56 (3.23)	4.57 (3.17)	366.99 (34.95)	373.05(56.28)
		**N2 (Fz)**	-5.20 (2.97)	-4.31 (2.57)	248.63 (26.86)	260.42(25.98)

# there is no latency measure of the CNV

For the amplitude of the cue CNV, a significant group-by-task interaction emerged [z = 2.06, p = 0.04] with the ADHD group decreasing and the control group increasing in amplitude in the flanker version. We obtained a trend in the predicted direction for a difference between the groups in amplitude of the cue CNV in the CPT-OX [z = 1.66, p = 0.09, d = 0.57] and a significant group difference in the flanker version [z = 3.32, p < 0.001, d = 1.00], indicating that cognitive preparation processes are abnormal in the ADHD group for the flanker task. The difference between the groups is particularly visible in Figure 1. However, we did not observe a significant difference in the CNV amplitude between the tasks for either controls [z = -1.55, p = 0.12, d = 0.21] or probands [z = 1.36, p = 0.17, d = 0.31].

**Figure 2:**
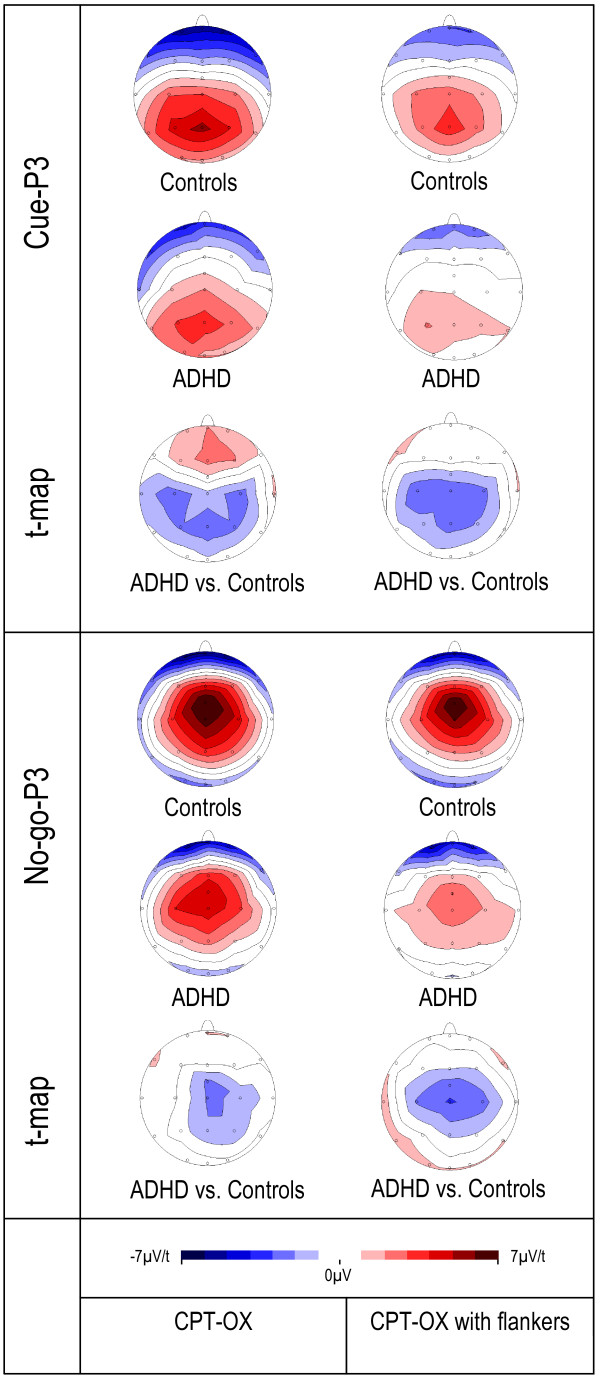
**Isocontour maps derived for the grand-average at the peak latency of the cue-P3 and the no-go-P3 for each group, plus t-maps for the group comparison**.

The analysis indicated a significant effect of age on the amplitude of the cue-P3 [z = -2.89, p = 0.001] yet no significant group-by-task interaction [z = -0.01, p = 0.99]; however, as predicted, the groups differed significantly for both tasks [z = -3.42, p = 0.001, d = 0.99]. This difference is particularly visible in Figure 2. A difference in cue-P3 amplitude between the tasks was significant, with both groups showing a decrease in amplitude in the CPT-OX [z = -3.39, p = 0.001, d = 0.46]. For the no-go-P3 amplitude there was no significant group-by-task interaction [z = -1.27, p = 0.20] but the groups differed significantly from each other [z = -2.49, p = 0.01, d = 0.73]. Figures 1 and 2 clearly show the attenuated no-go-P3s for the ADHD group in both tasks. We did not obtain significant differences between the tasks in no-go-P3 amplitude [z = -0.90, p = 0.37, d = 0.13]. The analysis of the amplitude of the P3 to go stimuli indicated that age was a significant covariate [z = -3.00, p = 0.003] yet no significant group-by-task interaction [z = 1.36, p = 0.17], group differences in go-P3 amplitude [z = -1.68, p = 0.10, d = 0.36] or difference in amplitude between the tasks [z = 0.14, p = 0.88, d = 0.21].

Spearman's correlations indicated significant association between the cue-P3 and the inhibitory P3 in the control group for the CPT-OX task and in both groups for the flanker version [CPT-OX: Controls: r = 0.46, p = 0.04; ADHD: r = 0.20, p = 0.40; CPT-OX flanker: Controls: r = 0.45, p = 0.04 ADHD: 0.60, p < 0.009]. Additionally, in order to test whether this association was specific to the cue and no-go-P3, Spearman's correlations were calculated between these components and the go-P3. These analyses indicated that there was not a significant relationship between the go and no-go-P3 components except for trend for the ADHD group in the flanker version [CPT-OX: Controls: r = 0.31, p = 0.19; ADHD: r = 0.27, p = 0.27; CPT-OX flanker: Controls: r = 0.17, p = 0.47; ADHD: r = 0.47, p = 0.05] and that there was a significant relationship between the cue-P3 and the go-P3 for both groups and both tasks [CPT-OX: Controls: r = 0.56, p = 0.01; ADHD: r = 0.65, p = 0.004; CPT-OX flanker: Controls: r = 0.56, p = 0.01; ADHD: r = 0.65, p = 0.004]. To further examine the relationship between motor preparation and response execution control, we additionally tested the relationship between the go and no-go P3 components and the CNV. No significant relationships emerged between the CNV and the go P3 component for either task in cases [CPT-OX: r = 0.14, p = 0.96; CPT-OX flanker: r = -0.02, p = 0.95] or controls [CPT-OX: r = -0.08, p = 0.74; CPT-OX flanker: r = -0.42, p = 0.86]. Similarly, no significant correlations emerged between the CNV and the no-go-P3 in the CPT-OX for either cases [r = 0.19, p = 0.43] or controls [r = -0.34, p = 0.17]; however a significant correlation did emerge in the CPT-OX flanker for cases [r = -0.52, p = 0.03] and a trend emerged for controls [r = -0.41; p = 0.08].

For the no-go-N2, the group-by-task interaction was not significant [z = -0.57, p = 0.57] and the groups did not differ significantly in no-go-N2 amplitudes [z = 1.62, p = 0.11, d = 0.47]. However, a significant difference emerged between the tasks [z = -2.48, p = 0.01, d = 0.33], with both groups showing an increase in amplitude in the flanker version. The group-by-task interaction was not significant for the go-N2 amplitude [z = -1.01, p = 0.31], but a group difference indicated a trend [z = 1.71, p = 0.08, d = 0.42] for an attenuation in the ADHD group. The tasks did not differ significantly in the amplitude of the go-N2 [z = -1.04, p = 0.30, d = 0.18]. The go and no-go-N2 components were significantly correlated for both groups and both tasks, though with the correlation for controls in the flanker version showing a trend only [ADHD: CPT-OX: r = 0.70, p < 0.004; CPT-OX flanker: r = 0.52, p = 0.02; Control: CPT-OX: r = 0.48, p = 0.03; CPT-OX flanker: r = 0.44, p = 0.07].

## Discussion

In a comparison of 21 male DSM-IV AD-ADHD cases and 20 age and gender matched controls, we identified a similar pattern of altered cognitive-electrophysiological processing to that previously reported in childhood ADHD. This finding suggests persistence of the abnormalities in underlying processes and provides external validation for the diagnostic construct of AD-ADHD.

Attenuation of the fronto-central no-go-P3 indicated the presence of abnormal inhibitory processing and confirmed previous ERP findings on AD-ADHD [23,25,26] and is in agreement with recent neuropsychological studies of AD-ADHD [e.g. 49]. Significant differences between the groups in the no-go-P3 amplitude, without a significant increase in the number of commission errors in AD-ADHD participants highlights the ability of electrophysiological measures to detect group differences in neuronal function in the absence of a large effect in performance differences.

The AD-ADHD group also displayed reduced cue-P3 amplitudes, indicating reduced attentional orienting to cue stimuli; and reduced CNV, indicating abnormal anticipation and preparation. This novel finding indicates that, similar to childhood ADHD [16-18,22,24,50], these brain electrical precursors of inhibitory processing are abnormal in AD-ADHD and confirms the recent finding of an association with ADHD symptoms in adulthood and CNV amplitude reduction [23]. The effect sizes for these cue processing deficits were larger in the flanker version of the CPT-OX, significantly so for the CNV. This is likely to partly explain the difference between our findings and those of Dhar et al., (2010), which did not indicate any cue processing deficits in AD-ADHD [29] but further differences are probably due to the calculation of current source densities in their analyses, as opposed to the traditional voltage potentials that we've used here. As the cue-P3 indexes the preparatory state elicited by orienting to a cue stimulus, we hypothesised that there would be a positive relationship with the subsequent process of response inhibition. Indeed, there was a significant correlation between the cue-P3 and the inhibitory P3 in the control group for the CPT-OX and for both groups in the CPT-OX with flankers. The correlation between these processes suggests that reduced allocation of resources to the cue stimulus, and therefore reduced expectancy for the upcoming go/no-go stimulus, is associated with reduced strength in inhibitory processing [17,45,51]. Importantly, there was no evidence for a relationship between the inhibitory P3 and another topographically distinct P3 component, the go-P3. This indicates that go and no-go-P3 reflect different processes but both are influenced by preparatory processes reflected in the cue-P3. However, the fact that the (intervening) CNV amplitude correlated only with the no-go-P3 suggests that abnormalities in the general allocation of attentional resources in ADHD is contributing to the response inhibition deficit. This finding could suggest a common influence of motivation and arousal on these processes [52] or less efficient signal conduction across the synapses [53]. Based on the current findings, it is unlikely that a primary deficit of response inhibition is an adequate explanation for the observed pattern of cognitive and behavioural impairments. As predicted, effect sizes for the flanker version of the CPT-OX were larger, indicating the increased sensitivity of this task to the processing deficits underlying AD-ADHD.

Despite a strong positive relationship between the cue-P3 and the go-P3 for both groups in both tasks, there was limited evidence of abnormal processing to stimuli associated with response execution control, as indexed by the go-P3. This is similar to findings in childhood ADHD that used the same task [16,17] and may indicate that ADHD-related attentional processing deficits are reduced by valid cues, regardless of abnormal cue processing. It may also indicate that go P3 deficits in ADHD are related to state regulation, as decreased go P3 components in ADHD are evident only in slow conditions [28,54].

In additional investigations we did not find abnormal go or no-go-N2 effects in AD-ADHD, which again was identical to previous findings in children with ADHD using this task [16,17,30]. We obtained significant positive correlations between the go and no-go-N2 components for both tasks and in both groups, suggesting that, in this task, the go and no-go-N2 partly reflect the same process, rather than the no-go-N2 representing a separate measure of inhibitory processing. Previous studies [50] reporting an attenuated no-go-N2 in ADHD used tasks other than the CPT-OX (e.g. the stop task), which possibly require an increased level of conflict monitoring. The no-go-N2 in the current CPT is correlated with a prepared response, whereas in the stop task it is correlated with an ongoing response that has to be inhibited [50].

In terms of task performance, there was no evidence of speed-accuracy trade-off differences between the groups and adults with ADHD were slower with more variable RTs than controls. In particular, RT variability across a wide variety of neuropsychological tests was recently reported to be the best discriminator between ADHD cases and controls, with principal components analysis suggesting that RT variability across tasks forms a unitary construct [55]. Furthermore, alterations in factors such as rewards [56] or combined reward and faster event rate [57] can lead to greater improvement in RT variability for ADHD cases than controls (see also [58]). One potential explanation for the overall pattern of findings (abnormal preparatory states elicited by cues and correlated with inhibitory responses; and the association of ADHD with RT variability and the interaction with event rate and reward of stimuli) is a more general state regulation deficit [59] indicated by reduced cortical arousal in ADHD. This should be directly tested in future research. The finding of an increased EEG theta/beta ratio, indicating low arousal in ADHD across the lifespan [60], is consistent with this proposal. The recent finding of an association between increased theta activity (indicating underarousal) and RT variability in childhood ADHD provides direct support for this hypothesis [61] as does another recent investigation showing a relationship between a reduction in arousal over time and increased reaction time variability [62]. RT variability is not specific to ADHD, but is observed also in other disorders including autism [e.g.63] and schizophrenia [64,65]; yet we do not know whether the underlying causes are shared or disorder-specific.

To increase homogeneity of the sample, the study included only males; however a recent study confirmed the findings presented here in females [23]. To maximise the homogeneity of the sample and minimise the impact of potential confounding conditions, the participants with ADHD were selected to have no major comorbidities. This highly selected group had slightly higher than expected IQs, however, the ADHD group was well matched with the controls for IQ. To test the generalisation of these findings to more typical clinical samples, future studies should include individuals with a wider range of IQs. Further, to test if these processes are developmentally stable, longitudinal studies are required that investigate these processes from childhood to adulthood, incorporating adults who do not retain the ADHD diagnosis.

## Conclusions

In conclusion, in a detailed combined investigation of ERP indices of response execution, inhibition and preparatory processes in AD-ADHD, we found a similar profile of altered processing deficits as previously identified in children with ADHD. This suggests that the ERP measures represent underlying processes that are developmentally stable. Furthermore, these findings provide external validation of the ADHD diagnosis in adults, which is becoming increasingly recognised as a common psychiatric disorder in adulthood [3,8]. Since the ERP indices were impaired even in the absence of performance deficits, and ERP variables have been shown to be reliable and heritable indices of brain function, they may be particularly useful in studies that aim to further our understanding of the processes that mediate genetic influences on behaviour. Further research that links these techniques to other indices of brain function, such as measures of arousal states or localizing data from functional magnetic resonance imaging, may therefore help in the development of causal models of ADHD that link genetic and environmental variation throughout the lifespan, to functional differences in molecular biology, physiology, and behaviour.

## Competing interests

The authors declare that they have no competing interests.

## Authors' contributions

GM carried out the EEG assessments, performed the data analysis and drafted the manuscript. BA, TB, DB and AR provided training and supervision in EEG analysis. PA and JK conceived of the study, participated in its design and coordination and helped to draft the manuscript. All authors read and approved the final manuscript.
